# The Influence of Caffeine Expectancies on Simulated Soccer Performance in Recreational Individuals

**DOI:** 10.3390/nu11102289

**Published:** 2019-09-25

**Authors:** Akbar Shabir, Andy Hooton, George Spencer, Mitch Storey, Olivia Ensor, Laura Sandford, Jason Tallis, Bryan Saunders, Matthew F. Higgins

**Affiliations:** 1 Human Sciences Research Centre, University of Derby, Kedleston Road, Derby DE22 1GB, UK; a.shabir2@derby.ac.uk (A.S.); a.hooton@derby.ac.uk (A.H.); g.spencer5@derby.ac.uk (G.S.); m.storey2@unimail.derby.ac.uk (M.S.); o.ensor1@unimail.derby.ac.uk (O.E.); l.sandford1@unimail.derby.ac.uk (L.S.); 2 Centre for Applie and Biological and Exercise Sciences, Coventry University, Priory Street, Coventry CV1 5FB, UK; ab0289@coventry.ac.uk; 3 Applied Physiology and Nutrition Research Group, School of Physical Education and Sport, Rheumatology Division, College of Medicine FMUSP, University of Sao Paulo, Sao Paulo, SP 05508-030, Brazil; drbryansaunders@outlook.com

**Keywords:** sport, exercise, expectancy, belief, perceptions, placebo effect

## Abstract

Caffeine (CAF) has been reported to improve various facets associated with successful soccer play, including gross motor skill performance, endurance capacity and cognition. These benefits are primarily attributed to pharmacological mechanisms. However, evidence assessing CAF’s overall effects on soccer performance are sparse with no studies accounting for CAF’s potential psychological impact. Therefore, the aim of this study was to assess CAF’s psychological vs. pharmacological influence on various facets of simulated soccer performance. Utilising a double-dissociation design, eight male recreational soccer players (age: 22 ± 5 years, body mass: 78 ± 16 kg, height: 178 ± 6 cm) consumed CAF (3 mg/kg/body mass) or placebo (PLA) capsules, 60 min prior to performing the Loughborough Intermittent Shuttle Test (LIST) interspersed with a collection of ratings of perceived exertion (RPE), blood glucose and lactate, heart rate and performing the Loughborough Soccer Passing Test (LSPT). Whole-body dynamic reaction time (DRT) was assessed pre- and post- LIST, and endurance capacity (T_LIM_) post, time-matched LIST. Statistical analysis was performed using IBM SPSS (v24) whilst subjective perceptions were explored using template analysis. Mean T_LIM_ was greatest (*p* < 0.001) for synergism (given CAF/told CAF) (672 ± 132 s) vs. placebo (given PLA/told PLA) (533 ± 79 s). However, when isolated, T_LIM_ was greater (*p* = 0.012) for CAF psychology (given PLA/told CAF) (623 ± 117 s) vs. pharmacology (given CAF/told PLA) (578 ± 99 s), potentially, via reduced RPE. Although DRT performance was greater (*p* = 0.024) post-ingestion (+5 hits) and post-exercise (+7 hits) for pharmacology vs. placebo, psychology and synergism appeared to improve LSPT performance vs. pharmacology. Interestingly, positive perceptions during psychology inhibited LSPT and DRT performance via potential CAF over-reliance, with the opposite occurring following negative perceptions. The benefits associated with CAF expectancies may better suit tasks that entail lesser cognitive-/skill-specific attributes but greater gross motor function and this is likely due to reduced RPE. In isolation, these effects appear greater vs. CAF pharmacology. However, an additive benefit may be observed after combining expectancy with CAF pharmacology (i.e., synergism).

## 1. Introduction

Caffeine (CAF) is the most frequently used psychoactive substance in sport, and has been observed to improve various exercise modalities that may benefit soccer performance including: strength and power output [1,2] endurance capacity [3,4,5] and gross motor skill performance [6,7,8]. Caffeine’s ergogenic effects are typically observed with oral doses between 3–9 mg/kg/body mass (BM), with its most commonly associated mechanism ascribed to the blockade of adenosine receptor sites and subsequent central nervous stimulation [9,10].

Caffeine’s stimulatory properties may improve soccer performance by ameliorating physical and/or cognitive fatigue, which has been observed to reduce the total distance ran (~5%–10%) and frequency of sprints (~3%–4%) between the first and second half of games [11,12,13,14,15,16]. Associatively, the majority of goals conceded are also within the latter stages of halves [17], specifically, between min 30–45 (18%) and 75–90 (23%) whereby physical and/or cognitive fatigue has likely peaked. In contrast, the least goals are conceded within min 0–15 (12%) and 45–60 (16%) when physical and/or cognitive fatigue is at its lowest or has been somewhat replenished during the half-time interval. However, studies directly assessing CAF’s influence on soccer performance remain scarce and those that have done so almost exclusively attribute any benefits to pharmacological mechanisms [3,4,6,7,8].

Shabir et al. [18] indicate the psychological permutations (e.g., changes in motivation, perceptual exertion, belief, mood states, etc.) associated with expectancy of oral caffeine consumption may influence sport, exercise and/or cognitive performance comparably or to a greater extent vs. CAF pharmacology [19,20]. Expectancy effects of varying magnitude were observed across 13/17 studies. Moreover, studies assessing sport and exercise performance were always influenced by expectancies. These effects were facilitated by various mechanisms including the perception of mild side effects and augmented physiological arousal [21,22,23], changes in mood states [21,24], reductions in perceived effort [22,25] and changes in motivation [21,26]. Moreover, in contrast to adenosine receptor sensitivity, expectancies/beliefs may be trained and/or manipulated, further enhancing any ergogenic experience. However, at present the influence of CAF expectancies remain generally unaccounted for across sport and exercise performance with no soccer-specific studies accounting for any potential effects. However, CAF supplementation in recreational sport is commonly achieved via off-the-shelf products (e.g., coffee, energy drinks etc.) many of which entail low CAF doses (likely lower than 3 mg/kg/BM in most cases), thus CAF-induced benefits here may already originate from expectancy rather than pharmacology. Furthermore, expectancies have been found to enhance attributes that may facilitate improvements in soccer performance, including lower limb strength/power output [19,21,22,25,27], endurance capacity [19,27,28,29], concentration [30], memory [31] and attentional focus [32]. Expectancies could also ameliorate the quality of exercise recovery, training, and preparation for sports competitions which may be impaired following CAF consumption prior to late evening games due to changes in melatonin production and molecular oscillations [33]. Moreover, regular CAF dosing (such as that which might be expected across the course of a season in soccer) may result in a reduced pharmacological effect due to habituation to CAF’s central effects [34,35,36] and this may be overcome if expectancy elicits an effect.

In order to validly compare CAF’s psychological vs. pharmacological influence on sport and exercise performance, participant beliefs should be intentionally manipulated in accordance with the experimental purpose. This reduces the discrepancy of individuals guessing which supplement they have ingested that if uncontrolled might cause overlaps between pharmacology and psychology, making it difficult to delineate the individual effects of these properties. The double-dissociation design is considered most suitable here [18] and includes four groups representing a placebo (given placebo (PLA)/told PLA (GP/TP)) and the pharmacological (given CAF/told PLA (GC/TP)), psychological (given PLA/told CAF (GP/TC)) and synergistic effect(s) of CAF (given CAF/told CAF (GC/TC)) on the dependent variable(s) assessed.

Thus, the novelty and purpose of this study was to explore CAF’s psychological vs. pharmacological impact on measures of simulated soccer performance (e.g., skill proficiency, dynamic reaction time (DRT), and endurance capacity) and perceptual states, prior to, during and following intermittent exercise replicating the metabolic demands of a 90-min soccer game [37]. We hypothesised, in comparison to a placebo (i.e., given placebo/told placebo), CAF’s isolated psychological and/or pharmacological impetus would improve all facets of soccer performance to a greater extent. Moreover, synergism of CAF psychology and pharmacology would instigate the greatest benefit, although CAF psychology would prove of greater efficacy vs. CAF pharmacology and any improvements would be driven by enhanced perceptions.

## 2. Methods

### 2.1. Participants

After obtaining institutional ethical approval (ethics code—39-1617-ASs), participants were emailed an information sheet including all relevant study specific information which was confirmed verbally before informed consent was provided. Participants were required to be healthy, non-smoking, recreational male soccer players, between 18–40 years old. Subsequently, eight male participants (age: 22 ± 5 years, body mass: 78 ± 16 kg, height: 178 ± 6 cm) completed this study. This sample size is similar to previous studies exploring the influence of CAF expectancies on sport and exercise performance [21]. Recreational participation was defined as involvement in soccer specific activities (e.g., 5, 8 and/or 11 aside soccer games) at an amateur standard for 1.5 h per week, across at least 6 months. Although habitual CAF consumption was not confirmed, beliefs regarding CAF ergogenicity were explored at various time points (Section 2.8).

### 2.2. Pre-Experimental Procedures

Participants completed physical activity readiness (PAR-Q) and blood-screening questionnaires prior to participation. Participants were required to avoid strenuous exercise and alcohol 24 h, and CAF 12 h, prior to all exercise trials [38,39,40]. All participants verbally confirmed that they were not using ergogenic aids at the onset of this study and were prohibited to do so during participation. Participants attended trials 2 h post-prandial and were asked to maintain the same diet 24 h prior. This was recorded via self-reported food diaries and checked visually (e.g., food items included within diet logs were examined and compared to logs obtained during previous trials to ensure replication) whilst participants also verbally confirmed the aforementioned prior to each session. To avoid the confounding influence of changes in macronutrient and/or energy availability, significant importance was placed on consuming the same meal prior to each session. Dependent on the time of trials, an ideal breakfast/lunch plan was outlined to assist participants replicating their diets. Subsequently, all participants replicated their diets prior to each experimental trial. Each participants’ trials commenced at the same time of day to avoid the influence of circadian changes on exercise performance [41].

### 2.3. VO_2MAX_ and Brief Familiarisation

This study entailed a within-subjects, counterbalanced, double-blind, double-dissociation, mixed methods design. Participants attended the laboratory on 6 separate occasions, with trials separated by at least 48 h recovery. Trial one (T1) involved ascertaining an estimate of maximal oxygen uptake (VO_2MAX_) via a 20 m progressive shuttle run test [42] similar to that used in Nicholas et al. [37], and familiarisation of the main experimental protocols adopted. Briefly, after 5 min seated rest, heart rate (HR; F1 Polar Heart Rate Monitor, Polar, Kempele, Finland) was telemetrically recorded, and a finger prick capillary blood sample was taken to later assess blood lactate BLa and glucose BG concentrations (Biosen C_line, EKF Diagnostic, Magdeburg, Germany). Blood was collected into a 20 μL sodium heparinised capillary tube (EKF diagnostics, Cardiff, United Kingdom) which was then added to a 1 mL Eppendorf tube and mixed well before being placed into the Biosen C-Line for analysis. The shuttle run test involved 20 m running bouts between two cones at increasingly fast speeds until volitional exhaustion. This was controlled by auditory beeps (20M Bleep Test; Version 2.1; developer: Adam Howard, United Kingdon, London, 2016) using a smart phone device connected to a large portable speaker. Volitional exhaustion was defined as an inability to reach two consecutive cones in the allotted time, or via voluntary stoppage. To stimulate maximum effort, participants were provided consistent verbal encouragement. Upon completion, HR, blood sampling, both as previously described and ratings of perceived exertion (RPE; 6–20 category scale [43]) were recorded. From this, running speeds corresponding to 55% and 95% VO_2MAX_ were calculated for subsequent use during the Loughborough Intermittent Shuttle Test (LIST) [37].

Following a further 45-min seated rest, participants completed familiarisation and a baseline session measuring DRT (see Section 2.6), before performing the Loughborough Soccer Passing Test (LSPT) as described in McGregor et al. [44]. Two consecutive 15 min bouts of LIST (e.g., repeated sequences of: 3 × walking, 1 × sprint, 3 × cruising (55% VO_2MAX_) and 3 × jogging (95% VO_2MAX_); Part A) were then performed, with each bout followed by recording RPE and HR, blood sampling and completion of the LSPT, prior to 3 min rest (N.B. bouts of LIST across all trials were followed by similar measurements). All bouts pertaining to part A were controlled using a LIST sequencer software package (Nottingham Trent University, Nottingham, Clifton, England). Part B (T_LIM_) (only relevant to trials 2 to 6, inclusive) was controlled manually using an online tone generator [45] and involved 20 m running bouts at 55% and 95% VO_2MAX_ until volitional exhaustion. Following completion of both 15 min LIST bouts, a second session measuring DRT was performed before participants left the laboratory.

### 2.4. Full Familiarisation and Experimental Trials

**Figure 1:**
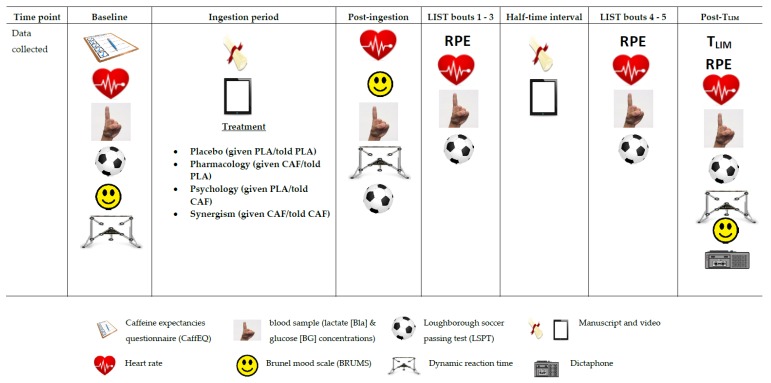
Experimental protocol outline for trials 2–6. Legend (N.B: Caffeine expectancies questionnaire (CaffEQ) and Dictaphone only utilised during trials 2, 3 and 5 and trials 4 and 6, respectively). (PLA = placebo; CAF = caffeine; RPE = ratings of perceived exertion [43]; LIST = Loughborough Intermittent Shuttle Test; CaffEQ = caffeine expectancies questionnaire [50].

An outline of the main methodological practices implemented during full familiarisation (T2) and experimental trials (T3–T6), can be found in Figure 1. Trials lasted approximately 4 h. Briefly, following 5 min seated rest, HR and a blood sample were taken to measure BLa and BG concentrations. Mood states were subsequently assessed using the Brunel Mood Scale (BRUMS; Section 2.7) [46]. Individuals then performed the LSPT, before familiarisation and a baseline session measuring DRT. This was followed by administration of 1/4 treatments (Section 2.5). Treatments were consumed within 5 min of a 60 min seated ingestion period [10], where participants rested quietly in a semi-supine position. Following this all baseline parameters were reassessed. After completing the LSPT, individuals then rested for 3 min before performing 3 consecutive bouts of the LIST. A 15 min break replicating the half-time interval during soccer games was implemented prior to bouts 4, and 5, followed by part B of the LIST. All measures following LIST were recorded for a final time, as were DRT and completion of the BRUMS.

During the full familiarisation session water intake was measured and replicated during experimental trials. Furthermore, at the start of familiarisation and experimental trials 1 and 3, participants completed the CAF expectancies questionnaire (Section 2.8) ((CaffEQ): 47) which aimed to assess habituated expectancies and whether expectancies changed between trials. Additionally, using a Dictaphone (Section 2.9), individuals recorded a short verbal description of their experiences at the end of experimental trials 2 and 4.

### 2.5. Treatments

Treatments involved oral consumption of visually identical PLA (3 mg/kg/BM cornflower) or CAF (3 mg/kg/BM) capsules and were always administered by a member of the technical support team who was otherwise uninvolved during data collection. We adopted the lowest typical ergogenic dose of CAF [10], as Goldstein et al. [5] observed no differences in sport and exercise performance between low to moderate (3–6 mg/kg/BM) doses. Furthermore, greater doses may induce debilitative side effects and, therefore, override CAF ergogenicity, for some individuals [47]. To facilitate expectancies for CAF ergogenicity a manuscript and brief video [48] highlighting CAF’s benefits on exercise performance were used for told CAF conditions. Contrastingly, the manuscript used during told PLA conditions was designed to invoke a neutral effect, whilst the video [49] was standardised to have minimal impact on perceptual states, or influence information relayed during told CAF conditions. These manuscripts/videos were re-administered within the first 5 min of the half-time interval. As such four treatments were administered across experimental trials: (1) placebo (given PLA/told PLA), (2) pharmacology (given CAF/told PLA), (3) psychology (given PLA/told CAF) and (4) synergism (given CAF/told CAF).

### 2.6. Dynamic Reaction Time (DRT)

Whole body dynamic reaction time was measured using the BATAK Pro (Quotronics Limited, Surrey, UK) and is considered an important component across various soccer skills including tackling and shooting [51,52]. Individuals were required to hit as many randomly illuminated targets as possible, within 60 s (s). To our knowledge there is currently no familiarisation data regarding DRT using the BATAK Pro; therefore, we adopted a comparable protocol to the Sport Vision Trainer which is validated in assessment of reliability and repeatability pertaining to hand–eye co-ordination [53]. The mean deviation in DRT scores were within ~1–2 hits across all experimental trials, suggesting participants were appropriately familiarised to this protocol. All experimental data is reported as the average of 2 × 60 s attempts (defined as one session), with each attempt separated by 1 min of seated recovery.

### 2.7. Brunel Mood Scale (BRUMS)

The BRUMS assessed participant mood states. The BRUMS consists of 24 items equally arranged into six subscales (anger, confusion, depression, fatigue, tension and vigour), and like all other perceptual measures employed, its purpose was explained, and demonstrated prior to use. Participants were required to rate each item on a subscale of ‘not at all’ to ‘extremely’ with each rating entailing a corresponding numerical, arbitrary unit (AU) (0 = not at all, 1 = a little, 2 = moderately, 3 = quite a bit, 4 = extremely). The sum of responses for each subscale was subsequently divided by 4 to provide a final score. The BRUMS has high reliability and validity, with details of its development and validation found in Terry et al. [54].

### 2.8. Caffeine Expectancies Questionnaire (CaffEQ)

The CaffEQ is a 47-item self-report questionnaire which assesses habituated expectancies across a range of subscales related to caffeine expectancies including: withdrawal/dependence, energy/work enhancement, social/mood enhancement, appetite suppression, physical performance enhancement, anxiety/negative physical effects, and sleep disturbances. The CaffEQ involved choosing a vehicle that best described individuals most commonly used CAF source(s). If participants were naive to CAF use, they were advised to base responses on their expectancies. Each item was evaluated on a scale of ‘very unlikely’ to ‘very likely’ with each rating ascribed a numerical value (0 = very unlikely, 1 = unlikely, 2 = a little unlikely, 3 = a little likely, 4 = likely, 5 = very likely) which was later analysed to provide a score for each corresponding sub scale. The CaffEQ represents good, internal consistency (0.88–0.96) and construct validity (0.80–0.94) [50].

### 2.9. Dictaphone

Using a standardised neutral script, participants were encouraged to record a verbal description (lasting up to 5 min) comparing their experiences at the end of experimental trials 2 and 4. Information reminding what perceived treatment participants had consumed was provided within an A4 sheet of paper which was folded to uphold confidentiality from the lead researcher. Specific importance was placed on individuals remaining honest and there being no right/wrong answer(s). Participants were instructed only to commence recording once they understood what was expected from them and not to share any information with the research team. Participants were then provided an opportunity to ask any questions before being left alone for recording to commence. A member of the technical support team later collected the Dictaphone. These recordings were only made available to the lead researcher following completion of data collection.

### 2.10. Qualitative Analysis

Following auditory transcription of Dictaphone logs, written data was explored by means of template analysis [55]. Template analysis provides flexible use of theoretical underpinnings from both content analysis [56] and grounded theory [57]. To facilitate template analysis, each transcription was explored thematically, in line with the phases outlined in Braun and Clarke [58]. The subsequent findings were, therefore, relative to the researcher’s interpretation of subjective quotes. Once a list of codes had been compiled for each participant, these were linked/and or differentiated to create themes. Moreover, in line with Jackson [59], the following three practices were implemented to enhance trustworthiness [60] and credibility [61] during analysis:

(1) An in-depth description of the data collection and analysis procedure.
(2) Involvement of A.H and M.F.H in guiding the qualitative process, by making implicit enquiries to the lead researcher (A.S) about the data collection/analysis procedure. This assisted in minimising biases, whilst improving the clarity of interpretations.
(3) Brainstorming of pre-existing ideologies associated with the phenomenon in question to ensure the researcher was cognisant of their own inherent beliefs and their influence upon the identification of codes, themes, and/or concepts [59,62].

Participant identity was protected by use of pseudonyms. However, to provide greater meaning to the qualitative findings, names were used as opposed to numbers.

### 2.11. Statistical Analysis

Quantitative statistical analysis was completed using IBM SPSS (v25 IBM Corp, Armonk, New York, NY, USA). For all data, normality (via Shapiro-Wilk’s test) and homogeneity of variance/sphericity (via Mauchly’s test) was checked. If sphericity was violated or data was non-normally distributed, degrees of freedom were corrected using Greenhouse–Geisser values or the appropriate non-parametric test was selected [63]. Confidence intervals were explored using least significant difference (LSD) (none) over Bonferroni corrections to minimise the potential of missing meaningful effects. The Bonferroni correction aims to reduce the chance of type 1 errors but subsequently increases the likelihood of type 2 errors and may be regarded a conservative approach that is better suited to experiments that have no clear hypothesis [64]. For analysis of variance (ANOVA, i.e., repeated measures) main effects and interactions, the effect size (ES) is reported as the partial η^2^ value. Otherwise, the ES (Cohens d) was calculated using the difference in means divided by the pooled standard deviation (SD) of the compared values for normally distributed data [65], and Z/√ n for non-normally distributed data [66]. Data is presented as mean ± standard deviation unless otherwise stated. The statistical threshold was set at *p* ≤ 0.05 [67,68].

## 3. Results

### 3.1. Endurance Capacity (T_LIM_)

**Figure 2:**
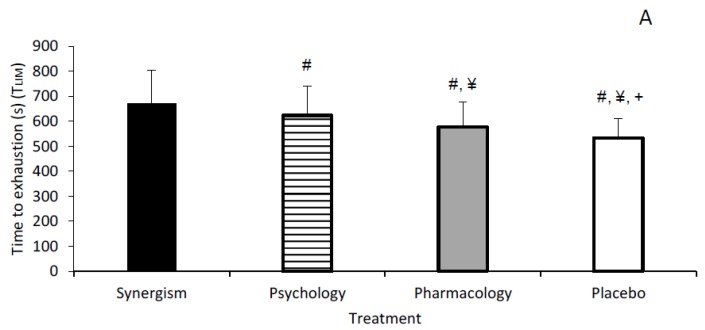

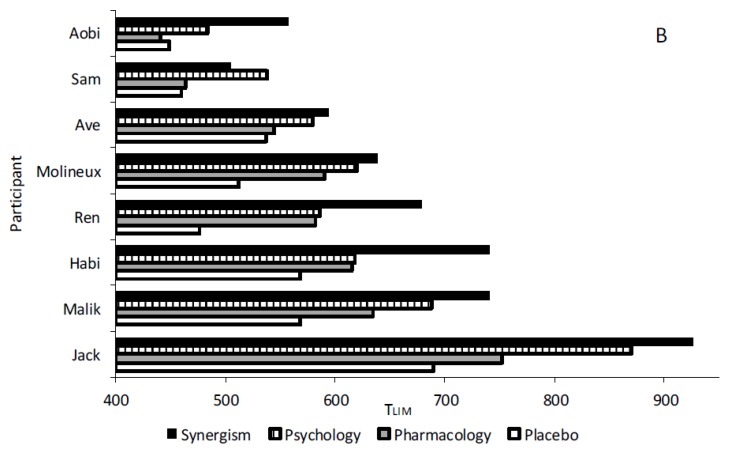
Endurance capacity (TLIM) scores (s). (**A**) Mean T_LIM_ (s) across treatments (#, ¥ and + denotes significantly lower vs. synergism, psychology and pharmacology, respectively); (**B**) subjective T_LIM_ across treatments.

There were no order effects for T_LIM_ (*p* = 0.485). A main effect for treatment was observed (*p* < 0.001; F = 23.638; η^2^ = 0.772). Mean T_LIM_ was greatest (*p* < 0.001) for synergism (672 ± 132 s) vs. placebo (533 ± 79 s) (Figure 2). However, when isolated, T_LIM_ was greater (*p* = 0.012; ES = 0.4) for psychology (623 ± 117 s) vs. pharmacology (578 ± 99 s) with all participants running longer for psychology (Figure 2).

Although main effects were observed for RPE, HR, BLa and BG across time (i.e., greater scores were observed for T_LIM_ vs. time matched exercise (isotime) (bouts of LIST)) with the exception of HR, no treatment or interaction effects were observed. However, when these measures at post-exercise were divided by each minute of T_LIM_, a trend of reduction was observed for synergism followed by psychology, pharmacology and placebo (Table 1 and Table 2).

### 3.2. Dynamic Reaction Time (DRT)

No treatment x time interaction was observed (*p* = 0.759; F = 0.561; η^2^ = 0.074) but main effects were detected for treatment (*p* = 0.024; F = 3.854; η^2^ = 0.355) and time (*p* < 0.001; F = 20.802; η^2^ = 0.748). Fatigue appeared to debilitate DRT performance (*p* < 0.05), with a mean reduction of between 4 to 7 hits following T_LIM_ vs.

**Figure 3:**
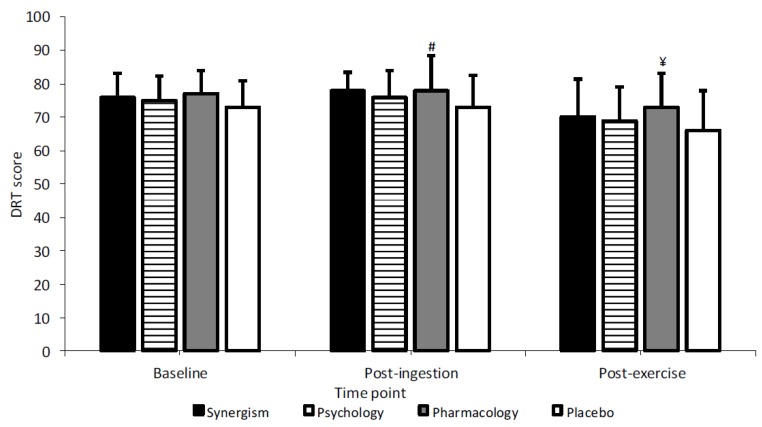
Mean dynamic reaction time (DRT) across treatments and time (# and ¥ denotes significantly greater difference vs. placebo).

Baseline and 5 to 9 hits vs. post-ingestion. However, pharmacology ameliorated this decline by 2 to 4 hits vs. all treatments. Individuals also achieved 5 hits more at post-ingestion (*p* = 0.05; ES = 0.5) and 7 hits more following T_LIM_ (*p* = 0.008; ES = 0.5), for pharmacology vs. placebo (Figure 3).

### 3.3. Loughborough Soccer Passing Test (LSPT)

**Figure 4:**
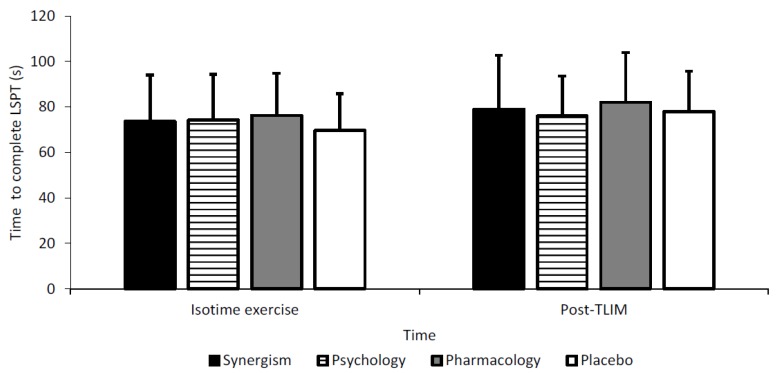
Time taken to complete the Loughborough Soccer Passing Test (LSPT) across treatments and time.

No interaction or main effects were observed across any LSPT parameter. However, time taken to complete LSPT following isotime exercise was fastest for placebo (70 ± 3 s) followed by synergism and psychology (74 ± 1 s) which were 2 s faster vs. pharmacology (76 ± 2 s) (Figure 4).

### 3.4. Heart Rate

No treatment x time interaction was observed for HR (*p* = 0.053; F = 1.613; η^2^ = 0.187), however main effects for treatment (*p* = 0.033; F = 5.359; η^2^ = 0.434) and time (*p* < 0.001; F = 1495.447; η^2^ = 0.995) showed greater overall HR for given PLA vs. CAF conditions and greater HR with increasing time.

### 3.5. Blood Variables

No treatment x time interaction or main effect for treatment was observed for BLa and BG. However, a main effect of time was detected for BLa (*p* < 0.001; F = 147.898; η^2^ = 0.967) and BG (*p* = 0.009; F = 3.281; η^2^ = 0.396) with BLa greater with increasing time, whilst BG was reduced.

### 3.6. BRUMS

No treatment x time interaction or main effect for treatment was observed for any BRUMS subscale. However, a main effect of time was detected for fatigue (*p* < 0.001; F = 51.501; η^2^ = 0.880) and vigour (*p* = 0.04; F = 14.587; η^2^ = 0.646). Generally, fatigue was greater with time, whilst vigour was reduced.

### 3.7. CaffEQ

Participant responses regarding caffeine expectancies entailed six independent modes of CAF consumption, with only Aobi representing more than one (Table 3).

No mean differences were observed between trials 1 and 3 across any CaffEQ subscales irrespective of the treatment administered. However, following subjective analysis various differences were observed across trials (Table 4).

## 4. Qualitative Findings

Following template analysis, 5 areas of discussion became prominent (general perceptions, DRT, LSPT, T_LIM_ and LIST; Table 5). Although the success of expectancy manipulation was not explicity confirmed, no participants correctly guessed the deception employed. Moreover, during Dictaphone use, Habi, Ren, Ave and Aobi referred to treatments as they were administered (i.e., told CAF/PLA), whilst Malik, Jack, Sam and Molineux referred to at least 2/4 treatments. Thus, it appeared participants believed the deception employed.

## 5. Discussion

Through implementation of a double-dissociation design, this study is the first to compare CAF’s pharmacological vs. psychological impact on various facets of simulated soccer performance. Although all treatments enhanced T_LIM_ vs. placebo, synergism resulted in the greatest improvements. However, when isolated, psychology improved T_LIM_ by 7% (~45 s) vs. pharmacology with all participants displaying improvements for psychology. These findings indicate CAF expectancy is an important contributor to the performance-enhancing benefit(s) of CAF. In relation to tasks involving a greater cognitive influence, pharmacology improved post-exercise DRT performance vs. all other treatments, whilst told CAF conditions improved the time taken to complete LSPT vs. pharmacology. Hence, CAF may be an effective nutritional supplement to evoke improved exercise performance. In some cases such benefits may occur with only the belief that CAF has been consumed and these effects may be greater vs. CAF’s pharmacology impetus. However, an additive effect may be observed after combining expectancy with CAF pharmacology [18].

Irrespective of the ingested treatment, expectancies improved T_LIM_ with psychology and synergism resulting in 90 and 95 s improvements vs. placebo and pharmacology, respectively. Using a double-dissociation model, only two other studies have explored the influence of CAF expectancies on T_LIM_, albeit during cycle ergometer based maximal incremental tests. Brietzke et al. [28] found synergism and psychology resulted in ~19% (~75 s) and ~17% (~68 s) improvements in endurance capacity vs. a control (i.e., no treatment administered; (CON)), whereas Pires et al. [29] observed ~15% (63 s) and ~17% (71 s) improvements vs. CON. Both studies utilised 6 mg/kg/BM CAF capsules, and recreationally active participants. Pires et al. [29] showed rectus femoris activation and pre-frontal cortex deoxygenation were augmented across both CAF treatments, vs. CON. The latter effect is associated with antagonism of A_1_ and A_2A_ adenosine receptors, and subsequent corticospinal excitability. Moreover, whilst Brietzke et al. [28] observed similar RPE for synergism and psychology, magnitude-based inferences indicated 75% probability of a beneficial effect for both conditions vs. CON. Comparably, we observed similar RPE across treatments following T_LIM_. However, when RPE was divided by T_LIM_, a trend of reduction was observed for synergism, followed by psychology, pharmacology and placebo. A similar trend was also observed for HR, BLa and BG. Hence, T_LIM_ performance was likely facilitated by lowered cardiovascular, hematological and/or perceptual strain, which appeared greater influenced by CAF expectancies vs. pharmacology. In support, Benedetti et al. [69] advocate that expectancies could influence changes in physiological processes associated with perceptual, motor, and homeostatic relevance. Furthermore, the psychobiological model of endurance performance posits that interventions designed to reduce perceptual exertion and/or enhance motivation may improve exercise tolerance [70,71]. Indeed, placebos have been observed to increase frontal alpha asymmetry and associated positive affect appraisal of effort perception, when described as ergogenic aids [72]. It is also plausible that perceptual exertion and/or motivation may share an inverse relationship [73], though subjective motivation was not directly assessed here. In contrast to the current study, the aforementioned studies were performed single-blind (i.e., potentially influenced by experimenter bias), whilst subjective perceptions were unexplored which are important in advocating CAF’s mechanisms of action [18].

Pharmacology resulted in five and seven score improvements during measurement of DRT, at post-ingestion and post-exercise, respectively, vs. placebo. Synergism also improved DRT at post-ingestion by 5 scores vs. placebo, thus CAF possibly facilitated augmented performance via central effects [74]. Moreover, the decline in DRT performance observed at post-exercise vs. baseline and post-ingestion was also ameliorated during pharmacology, with scores 2 to 4 and 2 to 3 hits greater vs. all other conditions. In contrast, Oei and Hartley [31] detected comparable performance on a self-designed sustained attention task for given CAF (~143 mg) (2.57 s) and told CAF (2.47 s) treatments. Moreover, similar findings were observed on the Bakan vigilance task for psychology, placebo and pharmacology (200 mg) [38]. The difference in results between the present study and the aforementioned studies may relate to the differences in tasks employed. Caffeine initiates excitability at the supraspinal level which may improve gross motor function (i.e., agility, reaction time, whole body movement) before, during and after sports activities [3,75,76,77]. In contrast, expectancy effects may be overestimated during the performance of simple reaction tests due to inhibition of fine motor skills associated with CAF over arousal and impaired cognitions [78].

Although the time taken to complete the LSPT declined over time, psychology and synergism appeared to mediate this following time matched exercise and T_LIM_, vs. pharmacology. These results were likely due to expectancies for CAF ergogenicity as performance was comparable for synergism and psychology. Gant et al. [62] reported CAF (3.7 mg/kg/BM) improved LSPT performance by 1.5 s following isotime exercise vs. CON, in 15 amateur male soccer players. Comparatively, Foskett et al. [7] observed a 2.3 s reduction for CAF (6 mg/kg/BM) vs. CON, across 12 university soccer players. Although neither study explored CAF’s psychological impact, Foskett et al. [7] found 4 individuals correctly, and 3 incorrectly, identified CAF trials with 5 declining to comment. Thus, although disparate, expectancies likely influenced these findings and this issue may be associated with a lack of double-dissociation design whereby expectancies were uncontrolled [18]. Moreover, expectancy effects are likely individually (based on belief and concurrent level of motivation), temporally and experientially modulated further highlighting the need to explore subjective perceptions. These issues may have also persisted in Gant et al. [79], although were not explored.

The changes in BG and BLa with increasing exercise intensity are likely causal and concomitant to augmented glucose metabolism associated with greater energy output and metabolite accumulation [80,81]. Furthermore, similar effects were observed for HR and are likely associated with a greater cellular requirement for oxygen and nutrients (e.g., glucose) and removal of metabolites and carbon dioxide [82]. Moreover, the 2 to 4 bpm^−1^ between-treatment variances in HR were likely physiologically negligible, especially as HR following isotime exercise was comparable across treatments (~164 bpm^−1^). These findings correlate with BRUMS, whereby fatigue increased and vigour decreased across time.

### 5.1. Qualitative Implications

#### 5.1.1. T_LIM_

The qualitative implications associated with T_LIM_ highlight the individualistic nature of subjective perceptions. However, told CAF treatments always facilitated greater or comparable T_LIM_ vs. told PLA and this was irrespective of whether perceptions for CAF ergogenicity were positive or negative [73]. For example, psychology was considered detrimental for Ren, yet T_LIM_ was comparable vs. pharmacology. Interestingly, Ren displayed expectancies for negative physical effects/anxiety but also performance enhancements on the CaffEQ, hence a relationship between these expectancies is plausible. Comparably, Ave documented significant fatigue perception across psychology and pharmacology, nonetheless T_LIM_ for psychology was comparable to synergism but 30 s greater vs. pharmacology. Molineux perceived minimal differences across treatments, though told CAF conditions performed comparably but ≥ 30 s vs. pharmacology. In contrast, Malik and Habi displayed limited expectancies across the CaffEQ, yet Malik felt psychology was the worst trial, whilst Habi indicated no differences. Interestingly, T_LIM_ was improved (53 s) or comparable vs. pharmacology, for Malik and Habi respectively. Thus, expectation of CAF consumption appeared to be the greatest mediating factor here. Furthermore, these findings are likely influenced by neutral expectancies and/or a lack of perceived effect for told PLA conditions. However, although the aforementioned was not confirmed, participants referred to treatments as they were administered (i.e., told CAF/PLA) and none guessed the deception employed.

#### 5.1.2. DRT and LSPT

The themes associated with DRT and LSPT appeared unrelated to performance outcomes. Instead, our findings indicate negative perceptions associated with CAF may invoke a greater cognitive impetus associated with alertness, concentration and technique which is otherwise impaired following positive perceptions due to CAF over reliance [23,26]. For example, for DRT, Aobi indicated psychology improved ‘reaction times’ on a day when he ‘wasn’t really feeling up to it’; however, 7 and 14 score reductions were observed vs. placebo at post-ingestion and post-exercise. Moreover, ‘the burst from the caffeine’ during synergism was also perceived to improve DRT, yet scores were comparable to placebo and 5 less vs. pharmacology, at post-ingestion. In contrast, Ren perceived greater fatigue for psychology vs. synergism, yet post-exercise DRT was 8 and 11 hits greater vs. synergism and placebo. Comparably, time to complete the LSPT was fastest for psychology vs. all other conditions after Malik felt the treatment impaired concentration, balance, motivation and technique. Opposingly, Aobi felt psychology was facilitative, yet LSPT performance was 7 to 12 s slower vs. all other conditions. This notion is supported by Tallis et al. [26] who propose an inverse relationship between expectations and motivation, with too positive an expectation resulting in reductions in conscious effort due to over confidence. We speculate similarly low expectancies associated with placebo may have driven improvements in LSPT due to increased conscious effort. However, greater clarity is required here, as limited subjective information was ascertained regarding placebo, following template analysis.

Although positive expectancies following psychology enhanced motivation, Harrell and Juliano [23] observed slower reaction times and less hits on the rapid visual information processing task vs. told impair conditions. Moreover, pharmacology appeared to improve performance vs. all treatments, irrespective of expectancies. Thus, much like the inverted U-hypothesis proposed by Yerkes and Dodson [83], expectations may need to be modulated to an optimal point for the greatest benefits and this point might differ individually (based on belief and concurrent level of motivation), temporally and experientially [18]. Given the potential difficulty in achieving this and the multi-faceted demands of soccer and other team sports activities, CAF expectancies might not be appropriate here given the potential for over-reliance with respect to cognitive-based tasks. Alternatively, CAF expectancies may better suit tasks that entail lower cognitive requirements but may benefit from improved gross motor function associated with reduced RPE (e.g., long-distance running, weightlifting etc.) [3,75,77].

### 5.2. Broader Applications

Although synergism of CAF psychology and pharmacology generally modulated the greatest performance benefits within the current study, when isolated, CAF’s psychological impetus appeared to mediate CAF ergogenicity to a greater extent vs. CAF pharmacology. Therefore, expectancies may represent an alternative to CAF dosing prior to late evening sports competitions, ameliorating the quality of exercise recovery, training and preparation which is otherwise impaired due to changes in melatonin production, molecular oscillations and sleep quality [33]. The aforementioned approach may also benefit soccer coaches in planning training sessions after accounting for variances in physical/mental recovery which would be aided by enhanced sleep quality. Moreover, these findings represent important implications for soccer players affected by habituation to CAF’s central effects [34,35,36] and health concerns (e.g., individuals suffering from heart disease, cardiac arrythmia, anxiety and depression) and side effects that are exacerbated/instigated by consumption of CAF and potentially detrimental to exercise performance [10,84,85,86,87]. Indeed, CAF expectancies represent minimal health concerns as the consumption of pharmacologically active CAF is not required. Moreover, during instances where CAF is consumed, expectancies may be trained and/or manipulated to enhance overall CAF ergogenicity (as indicated by the treatment ‘synergism’ during the current study). However, the influence of CAF expectancies has not been compared vs. CAF’s pharmacological effect following performance of subsequent games (e.g., soccer tournaments which are common across recreational sport). As such, it is unclear how CAF’s psychological effect would compare vs. CAF’s central effects here. Further research is required.

The current findings also emphasise the need for future CAF studies to account for any psychological effects which are at present largely overlooked. To achieve this, we recommend implementation of the double-dissociation design which involves manipulating beliefs in accord with the experimental purpose. This decreases the discrepancy of individuals guessing which treatment they have been administered and reduces overlaps between CAF psychology and pharmacology.

### 5.3. Limitations

Although no participants correctly guessed the deception employed, and treatments were generally referred to as they were administered (i.e., told CAF/PLA) we did not explicitly confirm the success of expectancy manipulation. Future research will benefit from confirming the success (or not) of expectancy manipulation.

We compared the subjective experiences of individuals via template analysis, however, CAF associated changes with respect to an individual’s circadian rhythm (i.e., changes in melatonin production and molecular oscillations) could have influenced these comparisons especially as some participants performed sessions in the morning, whilst others in the afternoon [33]. Moreover, subjective references were made to poor sleep quality possibly influencing exercise performance which may have been exacerbated by the timing of CAF consumption. Thus, future studies may benefit from measuring sleep quality prior to trials.

Although the notion of greater T_LIM_ associated with lowered RPE is supported by the psychobiological model of endurance performance [73], we did not measure subjective motivation which is also considered an important psychosomatic determinant of exercise tolerance. Future studies should, therefore, explore changes in motivation across treatments.

Although we explored changes in BLa and BG concentrations, CAF may also influence various other metabolites (e.g., epinephrine, norepinephrine etc.) [88,89] that might contribute to fluctuations in sport and exercise performance. Moreover, genetic assessments related to caffeine metabolism were not checked across participants which may have influenced the efficacy of CAF pharmacology [90,91].

Finally, while expectancies were assessed via the CaffEQ, we did not explore habitual CAF consumption, which has been observed to decrease the pharmacological effect of caffeine due to reduced adenosine receptor sensitivity, for habitual consumers [36]. Consequently, CAF’s psychological effect may have been overestimated across the current study. However, the effects of CAF withdrawal are likely minimal as generally participants did not indicate any withdrawal symptoms/sensations via template analysis or BRUMS. Moreover, it is unclear why we observed limited findings with respect of BRUMS, especially as various mentions were made to changes in mood states across all treatments, following template analysis.

## 6. Conclusions

Through implementation of a double-dissociation design, this study is the first to compare CAF’s pharmacological vs. psychological impact on various components of simulated soccer performance. Although all treatments enhanced T_LIM_ vs. placebo, synergism resulted in the greatest improvements. However, when isolated, psychology improved T_LIM_ by 7% (~45 s) vs. pharmacology with all participants displaying improvements for psychology. These findings appeared relative to enhanced expectancies and potentially reduced perceptual exertion but not perceptual states. Interestingly, DRT was impaired for individuals displaying positive CAF perceptions which may be explained by reduced conscious effort associated with CAF over-reliance. This was also observed during the LSPT with the opposite occurring during negative perceptions. Thus, the mechanisms by which expectancies influence exercise performance appear to be dependent on the task performed, with reduced RPE a potential key mediator during endurance capacity. Subsequently, CAF expectancies may better suit tasks that require lesser cognitive/skill specific attributes.

## Figures and Tables

**Table 1:** Post-exercise ratings of perceived exertion (RPE) divided by T_LIM_ per min (exercise termination across treatments advocated by *).

Treatment	1 min	2 min	3 min	4 min	5 min	6 min	7 min	Post-Exercise (Placebo)	Post-Exercise (Pharmacology)	Post-Exercise (Psychology)	Post-Exercise (Synergism)
Synergism	2	3	5	6	8	10	11	14	15	17	18*
Psychology	2	3	5	7	9	10	12	15	16	18*	-
Pharmacology	2	4	6	8	10	11	13	17	18*	-	-
Placebo	2	4	6	8	10	12	14	18*	-	-	-

**Table 2:** Post-exercise heart rate (HR), BLa and BG divided by T_LIM_ per min (exercise termination across treatments advocated by *).

Treatment	Post-Exercise (Placebo)	Post-Exercise (Pharmacology)	Post-Exercise (Psychology)	Post-Exercise (Synergism)
Heart Rate (HR; bpm^−1^)				
Synergism	147	159	172	185*
Psychology	160	173	187*	
Pharmacology	172	186*	-	-
Placebo	184*	-	-	-
Blood Lactate (BLa; mmol/L)				
Synergism	6.8	7.3	7.9	8.5*
Psychology	7.4	7.9	8.6*	
Pharmacology	8.4	9.0*	-	-
Placebo	8.6*	-	-	-
Blood Glucose (BG; mmol/L)				
Synergism	3.5	3.8	4.1	4.4*
Psychology	3.4	3.7	4.0*	-
Pharmacology	3.8	4.1*	-	-
Placebo	4.0*	-	-	-

**Table 3:** Beverage chosen during caffeine expectancies questionnaire (CaffEQ) responses.

Participant	Responses Based on
1-Jack	Caffeine in general
2-Malik	Energy drinks
3-Habi	Soft drinks
4-Ren	Energy drinks
5-Molineux	Other (not specified)
6-Ave	Caffeine in general
7-Sam	Energy drinks
8-Aobi	Coffee, soft drinks and tea

**Table 4:** Subjective CaffEQ scores across trials 1 and 3, alteration in expectancy type denoted by *. (i.e., 1 = unlikely, 2 = a little unlikely, 3 = a little likely, 4 = likely, 5 = very likely). T1 and T3 = trials 1 and 3.

	Withdrawal		Energy		Mood Enhancement		Appetite Suppression		Physical Performance Enhancement		Anxiety/Negative Physical Effects		Sleep Disturbances	
Participant	T1	T3	T1	T3	T1	T3	T1	T3	T1	T3	T1	T3	T1	T3
Jack	2	2	3	3	3	3	2	2	3	3	2	1	1	1
Malik	0	0	2	2	1	1	0	0	1	1	0	0	1	1
Habi	0	0	0	0	0	0	0	0	0	0	0	0	0	0
Ren	3	2 *	4	4	4	3	3	2 *	4	4	1	3 *	3	3
Molineux	2	3 *	2	3 *	1	2	2	3 *	3	3	2	3 *	1	2
Ave	0	1	2	2	1	1	0	3 *	2	3 *	0	1	0	2 *
Sam	1	0	3	2 *	1	1	0	1	2	3 *	0	0	0	0
Aobi	2	1	3	3	2	2	2	2	2	3 *	3	2 *	3	2 *

**Table 5:** Themes and supporting statements across areas of discussion.

General Perceptions	
Themes	Supporting Statements
Expectancies facilitated perceptions	Aobi—‘I felt like I needed the lift that day and you could definitely feel like the caffeine (trial—psychology) had an impact on me’ (greater mood and energy, and lowered fatigue perception vs. told PLA treatments).
	Ren—‘Compared to the two placebo trials, after the ingestion period (synergism), I almost immediately felt more alert, more active, more confident, and more energetic’. Synergism also reduced fatigue perception during LIST, vs. told PLA treatments.
	Ave—Had ‘a bit more energy’ for synergism vs. told PLA conditions.
Told PLA treatments had minimal effect	Aobi—Told PLA conditions induced neutral expectancies and/or a lack of ‘psychological effect’ and ‘didn’t really do much’
	Ren—‘I didn’t feel it had any effect on the (sic), obviously knowing it’s a placebo, both placebos (told PLA treatments), I expect what you’re expected to feel’
	Ave, Molineux and Habi indicated no differences between treatments.
**Dynamic reaction time (DRT)**	
Expectancies > told PLA treatments	Ren—Expected ‘to feel fatigued and slower’ during told PLA treatments prior to measurement of post-exercise DRT, whilst feeling quicker during synergism.
	Molineux and Aobi felt ‘more alert’ for psychology vs. placebo
	Aobi—Psychology improved ‘reaction times’ on a day when he ‘wasn’t really feeling up to it’.
	Ave—Told CAF conditions ‘really helped’, with synergism resulting in ‘a lot less misses’ and better performance vs. all other treatments
	Aobi—Felt more familiarised to complete DRT, however this was augmented by ‘the burst from the caffeine’ during synergism.
**LSPT and T_LIM_**	
Synergism > all other Treatments	Ave and Ren—Synergism improved LSPT vs. pharmacology Due to increased speed. Ren also felt he ‘was getting worse, getting a few more mistakes, missing the targets more’ during pharmacology.
	Aobi and Molineux were able to give more due to reduced fatigue perception for synergism vs. told PLA treatments, during T_LIM_.
	Ave—Synergism improved T_LIM_ vs. placebo due to reduced fatigue perception associated with ‘the caffeine’. However, ‘struggled’ more during psychology.
**LIST**	
Debilitative psychology	Malik put everything into LIST bout 1, and subsequently felt ‘fatigued’ and a ‘lack of motivation’ for psychology vs. told placebo treatments
	Ren—perceived greater cardiovascular and leg fatigue during psychology vs. pharmacology.
	Ave—felt tired during psychology but attributed this to a ‘lack of sleep’ and not the treatment.
	Ave and Molineux—no ‘improvement’ for psychology vs. placebo.

PLA = placebo; LIST = Loughborough Intermittent Shuttle Test; CAF = caffeine; LSPT = Loughborough Soccer Passing Test.
